# Future directions in the field of Bipolar Disorder

**DOI:** 10.1016/j.nsa.2024.104043

**Published:** 2024-04-23

**Authors:** E.Z. Reininghaus, N. Dalkner, L. Wels, M. Stijic, A. Reif, M. Manchia, O.A. Andreassen, ECNP Bipolar network, M. Bauer, A. Bechdolf, F. Bellivier, V. Bergink, D. Ceylan, C.U. Correll, B. Etain, A. Fagiolini, J. Geddes, A. Gonzalez-Pinto, G. Goodwin, T. Hajek, C. Henry, L.V. Kessing, R. Kupka, T.V. Lagerberg, M. Landen, R.W. Licht, M. Martinez-Cengotitabengoa, G. Morken, R.E. Nielsen, M. Rietschel, P. Ritter, T. Schulze, J. Scott, E. Severus, A. Squassina, A. Suwalska, E. Vieta, A. Yildiz

**Affiliations:** a Division of Psychiatry and Psychotherapeutic Medicine, Medical University of Graz, Austria; b Department of Psychiatry, Psychosomatics and Psychotherapy, University Hospital Frankfurt am Main - Goethe University, Frankfurt am Main, Germany; c Department of Medical Sciences and Public Health, University of Cagliari, Cagliari, Italy; d Department of Pharmacology, Dalhousie University, Halifax; e Canada Clinical Psychiatry Unit, University Hospital Agency of Cagliari, Cagliary, Italy; f NORMENT Centre, Division of Mental Health and Addiction, Oslo University Hospital, Oslo, Norway; g NORMENT Centre, Institute of Clinical Medicine, University of Oslo, Oslo, Norway

This special issue dedicated to the topic of bipolar disorder and its underlying structural, functional, psychological, and cognitive facets, as well as treatment, has been prepared by the European College of Neuropsychopharmacology (ECNP) Bipolar Disorders Network to summarize the results of a new format of standalone meetings of the ECNP Networks. This novel modality focused on a larger inclusion of early career clinical and translational scientists. In April 2023, the first ECNP Bipolar Disorders Network Standalone meeting with this new format (entitled “Bipolar Research bringing together the Generations”) took place in Frankfurt am Main (Germany). One early career scientist from each participating group could join the meeting and present their work at the meeting under the supervision of a network member. Twenty-four researchers in the field of bipolar disorder were present at the meeting (female n = 13, male n = 11), whereas ten early career scientists presented their work in a lecture as well as during a poster walk. Coordinated by the Bipolar Network, the presenters wrote a paper for this special issue on their research project presented at the standalone meeting.

Bipolar disorder is characterized by the occurrence of mood episodes in specific patterns over time, including depressive, mixed, hypomanic and manic episodes, which all present with pervasive and clinically relevant fluctuations in affective, cognitive-behavioural and neurovegetative symptom domains. While the risk factors include genetic susceptibility, as well as environmental factors such as stressful events in childhood, there is a large need for more knowledge about disease mechanisms to improve treatment.

Mikolas et al. (2024) could show in their review that *in vivo* exposure to stressful events in human subjects and the volumes of amygdala subregions are associated. The majority of the included studies found a negative association with stressful life events primarily within the basolateral complex of the amygdala as well as within superficial structures. The authors recommended that future, longitudinal studies should not only include young healthy individuals and subjects at risk for bipolar disorder, but also account for potential confounders, such as substance use and variation of body-mass index.

Klahn et al. (2023) observed that some individuals with bipolar disorder experience lingering symptoms and enduring impairments despite remission (i.e. euthymic phase). They explain this observation through aberrant functional connectivity of brain networks and report two abnormal connectivity markers in euthymic bipolar disorder: the first as an increased connectivity between temporal regions of the somatomotor and the frontoparietal network, while the second one is characterized by decreased connectivity within the somatomotor network and between the somatomotor and visual network. These findings might represent important targets for treatment of individuals with bipolar disorder.

Arat-Çelik et al. (2023) investigated neuropsychological aspects of bipolar disorder in relation to exposure to childhood trauma. They reported high prevalence of childhood trauma in individuals with bipolar disorder and their unaffected siblings. Both groups have experienced higher rates of emotional abuse and neglect, compared to healthy individuals. Nevertheless, neurocognitive impairments in the domains of global cognitive factor, processing speed, visual copying, verbal learning, and memory were observed only in the group of individuals with bipolar disorder. No significant impact of childhood trauma on neurocognitive domains was found.

Wels et al. (2023) focused on metacognitive aspects, namely cognitive insight and introspective accuracy in individuals with bipolar disorder. These abilities include how to understand and reflect upon one owns mental state and processes. The presence of bipolar disorder, however, can reduce these abilities and contribute to low compliance with treatment. Results of this study suggest that both cognitive insight and introspective accuracy should be interpreted as markers of affective symptomatic states rather than stable traits, which can predict the trajectory of illness and everyday functioning in individuals with bipolar disorder.

Finally, Völker et al. (2024), aimed to investigate the potential of saliva as a medium in monitoring lithium concentration to improve the quality of lithium medication. This is the gold standard in maintenance treatment of individuals with bipolar disorder I, but lithium treatment might be challenged by the need for continuous monitoring. The final objective of this project is to develop an easy-to-use point-of-care home testing device, which would increase safety and decrease or even eliminate the need for expensive laboratory analyses.

This special issue as the outcome of a new format of the standalone meetings of the ECNP Bipolar Network shows that including early career as well as senior scientists in such network meetings generates new and productive aspects. It is the ideal framework for personal exchange of projects and concepts with scientists of all generations. Future events or meetings of ECNP networks will invite young scientists to engage the next generation of bipolar disorder researchers in future directions of research and innovation on this challenging condition.

## Declaration of interests

The authors declare that they have no known competing financial interests or personal relationships that could have appeared to influence the work reported in this paper.
